# Environmental policy and equity prices

**DOI:** 10.1371/journal.pone.0289397

**Published:** 2023-07-28

**Authors:** Thorsten Lehnert

**Affiliations:** Department of Finance, University of Luxembourg, Esch-sur-Alzette, Luxembourg; Massey University - Albany Campus: Massey University - Auckland Campus, NEW ZEALAND

## Abstract

The information quality hypothesis suggests that, theoretically, the relationship between expected returns and conditional volatility is ambiguous and depends on the precision of the information signal, hence, it is affected by investors’ level of uncertainty. When investors’ uncertainty increases, the relationship may become negative. Using environmental policy as an imprecise signal of future economic performance, I find that a newspaper-based environmental policy-related uncertainty indicator (EPN) has a low correlation with equity market volatility, but has a significant negative impact on expected returns. A managed equity market portfolio that takes less (more) risk when the past EPN-related uncertainty is high (low) produces significant equity-risk-adjusted alphas. In particular, I show that EPN-timing is profitable, because it foresees the attractiveness of the mean-variance trade-off. Overall, an EPN-managed equity portfolio generates an annualized equity-risk-adjusted alpha of 5–6%. Interestingly, I find that the uncertainty around environmental policy is on average lower and, therefore, the strategy performs better during periods when the Republicans control the senate.

## Introduction

### US environmental policy

Environmental policy is primarily concerned with how to govern the relationship between humans and the natural environment in a mutually beneficial manner. In the US, environmental issues matter substantially in politics and penetrate the general consciousness enough to even affect elections. According to a recent survey by the Pew Research Center ([1], The Pew Research Center is a nonpartisan American think tank based in Washington, D.C. It provides information on social issues, public opinion, and demographic trends shaping the United States and the world), two-thirds of Americans still claim to experience the consequences of climate change in their local areas and think the federal government should do more to lessen these effects. Democrats as a whole (83%) believe that climate change has some or significant effects, while the majority of Republicans (62%) feel that climate change has little to no impact. Recently, in his column in The New York Times, which appeared in print on Aug. 16, 2022 with the headline “Why Republicans Turned Against the Environment”, Paul Krugman argues that “the gap has widened” and Republicans have become significantly less supportive of environmental action while Democrats have been even more so. He claims that “*environmental policy has been caught up in the cultural war*”. Indeed, for example in 1990, US Congress amended a very successful Clean Air Act with overwhelming, bipartisan majorities, taking action against ozone, urban smog, and acid rain among other things. The Kyoto Protocol, the first global agreement to reduce greenhouse gas emissions, was adopted in 1997. George W. Bush, however, withdraws the US from the Kyoto Protocol in June 2001 on the grounds that it exempts developing countries, like China, and would harm the US economy. Similarly, the first-ever universal, legally binding global climate agreement, the Paris Agreement, was adopted in December 2015. However, after an intense public discussion, President Donald Trump declared his intention to leave the Paris agreement in June 2017, which due to UN regulations took effect in November 2020.

### Climate change transition risks

According to the Network of Central Banks and Supervisors for Greening the Financial System [2], financial risks related to the environment can be divided into two categories: physical risks and transition risks. Extreme weather conditions and slow changes in climatic patterns are examples of how physical risks can have an impact on the financial sector, but the factors driving transition risks are more complex. First, companies that do not comply with the 2-degree scenarios may see their financial valuations decline and/or their credit ratings be downgraded as a result of policies related to climate change mitigation, such as the imposition of carbon pricing, because these companies have "stranded assets" that no longer generate an economic return on their prior investments, for instance because of the effect on their future discounted cash flows. For the financial institutions exposed to these carbon-intensive firms, this might also lead to market losses. Second, the relative pricing of alternative products and the market shares of specific enterprises could both be impacted by technical advancements, such as those assisting in the energy transition, which would lower profitability and eventually result in losses for financial institutions. Third, the economy and the financial system may be impacted by changes in public opinion, demand trends, and consumer preferences and expectations.

### Environmental policy news

In [3], the authors develop a newspaper-based indicator related to energy and environmental regulation that quantifies uncertainty around environmental policy (EPN). The authors analyze the articles’ content to evaluate the journalists’ assessments of the news stories, events, worries, and expectations that might influence equities return volatility. They find that policy news (tax, monetary, and regulatory policy) in general is a significant source of volatility, but EPN only contributes 1.3% to their overall news-based equity market volatility (EMV) measure. Interestingly, while EMV varies substantially with the VIX and the realized volatility of returns on the S&P 500, the respective correlation of EPN is quite low (22%). In contrast to other types of information mainly driving equity volatility, EPN measures concerns related to climate change, carbon tax, greenhouse gas, clean air and water, pollution controls etc. (see [3] for a complete set of specific terms). Hence, in the context of climate change and the low-carbon transition, EPN may offer a better measurement of transition risks.

**Fig 1:**
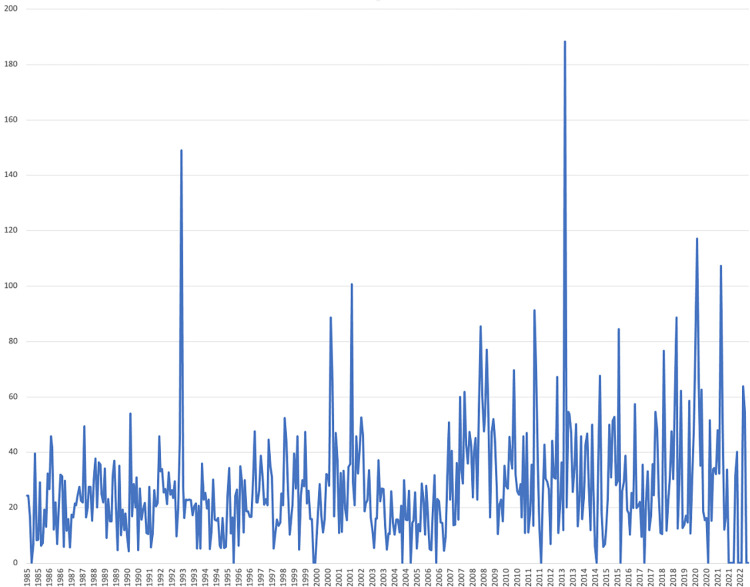
Time series of the environmental policy-related uncertainty indicator.

Fig 1 displays the resulting measure, which runs from January 1985 to October 2022. For example, a major spike occurred in February 1993 in reaction to the Clinton-Gore election and the related expected shift in US policy on global environmental issues. Another spike can be observed in December 2000 due to articles citing reports that the recent two-month period was the coldest on record in the US. While there was hope among US environmental advocates that a re-elected President Obama will give increased priority to climate protection, the most pronounced upward spike occurred in March 2013. The largescale damage, wide geographic scope of Hurricane Sandy, and the necessity for a multi-faceted emergency response seem to have catalyzed a change in US public opinion (see the Climate Alert of the Climate Institute for more detailed information). Additionally, the recent months were found to be one of the driest on record across the US. One of the recent spikes occurred in February 2020, where newspapers reported that the month marked the warm end to one of the most unusually warm months and winters on record for the globe.

### Environmental policy and equity prices

Typically, researchers empirically investigate the relationship between environmental policy and stock markets by using event study methodology, relying on policy announcements. In [4], the authors analyze the stock market reaction regarding a series of green policies following the signing of the Kyoto protocol in 1997. Overall, they find negative abnormal returns and an increase in systematic risk. While the effect is stronger for the biggest polluters, eco-friendly firms are less affected. Interestingly, they observe the particular pattern when President Obama took office after the 2008 US presidential election. In a related paper [5], the authors analyze a series of events, where after his election, President Trump initiated rollbacks of previously created regulations and policies. They find that Trump’s loosened environmental regulations and policies, which were initiated to strengthen the US economy, had only a minor impact. However, the coal industry benefitted from the policies experiencing recurring significant positive abnormal returns. In China, the recent enforcement of more stringent environmental regulation is related to obvious pollution problems. In [6], the authors analyze 10 environmental policies during 2014–2017. They find that heavily polluting firms consistently experience short-term negative stock market reactions following an announcement. In this paper, I make use of the methodology proposed in [7] to assess the economic significance of the impact of EPN-related uncertainty on aggregate stock returns. In [7], the authors show that a volatility-managed market factor generates positive alphas, increases Sharpe ratios and produces large utility gains for mean-variance investors. My EPN-related uncertainty-timing relies on a negative relationship between EPN-related uncertainty and expected return. Using a long time series starting in 1985, I document that indeed EPN predicts market returns and a related trading strategy generates positive equity-risk-adjusted alphas. A plausible motivation for my analysis is the information quality hypothesis of [8]. In the context of a dynamic asset pricing model with a representative agent, the author shows that the relationship between expected returns and conditional volatility is ambiguous and depends on the precision of the information signal of future economic performance, hence, investors’ level of uncertainty. When investors’ uncertainty increases (the external signal is not precise), the conditional expected excess return may become negative. Hence, volatility driven by learning about structural parameters might be priced differently than volatility driven by standard forms of risk. In [9], the authors demonstrate that, theoretically, low information-quality-driven volatility causes a negative connection between historical realized volatility and future projected volatility and returns. Their approach can explain a number of volatility-related asset pricing puzzles, including the realized volatility-timing results of [7]. Given that environmental policy can be considered an external imprecise signal rather than a standard form of risk, I hypothesize that an increase in environmental policy news contributes positively to investors’ uncertainty and, as a result, leads to negative expected returns.

The remainder of the paper is organized as follows. In the next sections, I sketch the Veronesi -type dynamic asset pricing model [8], describe the data and present summary statistics. In the subsequent sections, I present my empirical results and concludes with a summary of key results.

## Materials and methods

In a Lucas-type exchange economy [10], infinitely many identical investors have the following iso-elastic utility functions

$$u(c,t)=e^{-\phi t}\frac{c^{1-\gamma }}{1-\gamma }$$

where *ϕ* is the discount rate and *γ* the relative risk aversion coefficient. Investors’ opportunity set includes a bond that pays the risk-free rate of return *r* and a risky asset with a stochastic dividend *D* that follows the process

$$dD=\theta Ddt+\sigma_{D}DdB_{D}$$

where *B*_*D*_ denotes a standard Brownian motion. It is assumed that investors only observe a ‘noisy signal of the fundamentals’, but not the true time-varying drift *θ*(*t*)

$$de=\theta dt+\sigma_{e}dB_{e}$$

where *B*_*e*_ is another (*B*_*D*_-independent) standard Brownian motion.

The precision of the external signal is reflected in the inverse of the diffusion parameter he=1σe. Standard models with precise signals and constant drift *θ* assume *h*_*e*_ to be infinitely high. Alternatively, the precision of the noisy dividend signal is hD=1σD.

In the following, equilibrium prices are determined by standard market clearing conditions. [8] shows that in equilibrium the excess return process and its volatility can be derived

$$dR=\mu_{R}dt+(\sigma_{D}+h_{D}V_{\theta })\sigma_{e}d{\tilde{B}}_{D}+V_{\theta }h_{e}{\tilde{B}}_{e}$$

$$\sigma_{R}^{2}=\sigma_{D}^{2}+V_{\theta }[2+(h_{e}^{2}+h_{D}^{2})V_{\theta }]$$

where $\mu_{R}=\gamma (\sigma_{D}^{2}+V_{\theta })$. *V*_*θ*_ can be further defined, but, intuitively, characterizes investors’ uncertainty about the true growth rate of the economy and its own valuation of the asset. It can be shown that an increase in uncertainty corresponds to an increase in *V*_*θ*_ in absolute terms. Finally, one can derive the corresponding relationship between the conditional risk premium and the conditional variance $\mu_{R}=\gamma \sigma_{R}^{2}-{\gamma V}_{\theta }[1+(h_{e}^{2}+h_{D}^{2})V_{\theta }]$. Apparently, this relationship is ambiguous and biased by investors’ uncertainty through *V*_*θ*_. Hence, when risk-aversion is high and external signals of future economic performance imprecise, the conditional expected excess return may become negative when investors’ uncertainty increases. My main assumption regarding the empirical analysis in the subsequent sections is that information around environmental policy can be considered to be noisy signal on the future “health” of the economy. In general, policymakers are particularly interested in the relation between environmental policies and future economic performance. In [11], the authors critically review the empirical evidence. The authors argue that environmental policies are typically seen as a burden on economic activity, as they do not immediately increase output, but have a negative effect on costs. However, according to the ‘Porter Hypothesis’ appropriate environmental policies could inspire innovation, improves profitability and productivity, which as a result lowers the costs. In [11], the authors show that the empirical evidence is mixed and does not allow credible conclusions. The potential problem is that empirical studies are fragile and context-specific, and experience some data, measurement and estimation issues. In line with the idea of [8], I do not hypothesize if the economic performance is better under e.g. one political party’s majority, but what kind of effect the investors’ environmental policy-related uncertainty (the noisy signal) has on stock market prices.

My main variable of interest is a monthly newspaper-based indicator related to energy and environmental regulation that quantifies uncertainty around environmental policy (EPN). I also make use of text-based measures of uncertainty that are constructed using news on aggregate regulation (REG) and financial regulation (FINREG), as well as the broader equity market volatility (EMV) indicator derived in [3] that tracks the aggregate volatility in the US stock market for a period January 1985 until October 2022 (data are from the Federal Reserve Economic Data (FRED) database). In line with asset pricing theory, forward-looking measures of uncertainty based on options implied information can also be expected to be strong predictors of equity market returns. Research suggests that options contain information that relate to fluctuations in expected equity market volatility [12], in the variance risk premium (e.g. [13, 14]) or the likelihood of rare disasters (e.g. [15–17]). Times of relatively high expected risks, measured by risk-neutral volatility indices like VIX, are followed by above average excess returns on the market portfolio. Empirically, however, the ability of e.g. the VIX to predict returns is statistically rather weak. The results of [18] suggest that the relationship is nonlinear, which partly explains why VIX does not forecast stock returns significantly at any horizon.

Evidently, a time-series uncertainty- or volatility-based trading strategy might exploit an anomaly documented in the cross section. In order to exclude risk-based explanations, I also make use of the Fama-French factors and a momentum factor typically used in similar studies investigating the times series of S&P500 aggregate returns (the factors are from Kenneth French’s data library). Additionally, I make use of two Betting-Against-Beta (BAB) factors, the Novy-Marx and Velikov version [19] of the original BAB factor (EWBAB), as well as their modified version (VWBAB), constructed using more standard procedures (I thank Mihail Velikov for sharing the series with me). The authors show that the performance of the original BAB factor is driven by non-standard procedures used in its construction that actually equal weight stock returns. The correlation of the [19] version of the original betting-against-beta factor (EWBAB) with the [20] BAB factor, currently maintained by AQR (see https://www.aqr.com/Insights/Datasets/), is 99%, while it is 56% for their modified version (VWBAB), constructed using more standard procedures. Table 1 presents the summary statistics of the various variables used in the study.

**Table 1:** Summary statistics.

	Mean	Median	Std.	Min	Max
*MktRF*	0.73	1.19	4.53	-23.24	13.65
*SMB*	0.05	0.09	3.05	-17.29	21.48
*HML*	0.17	-0.02	3.14	-13.97	12.75
*MOM*	0.52	0.63	4.50	-34.30	18.20
*EWBAB (1985*:*01–2019*:*12)*	0.90	1.15	3.67	-17.44	15.72
*VWBAB (1985*:*01–2019*:*12)*	0.45	0.60	4.27	-17.45	16.41
*VXO/VIX (1986*:*01–2022*:*10)*	20.11	18.53	7.76	9.51	61.41
*EMV*	19.90	17.74	8.14	8.03	69.84
*REG*	4.98	4.51	2.12	1.57	21.17
*FINREG*	2.75	2.48	1.48	0.29	14.57
*EPN*	28.42	24.22	19.96	4.20	188.33

The table presents the summary statistics of the market excess return, the other factor portfolio returns, option-implied volatilities and news-implied uncertainty measures used in the study. The full sample period ranges from January 1985 to October 2022, covering a total of 454 months. MktRF is the excess market return, the value-weighted returns of NYSE, Amex, and Nasdaq stocks from the Center for Research in Security Prices (CRSP) over 30-day T-bill return in %. Other factors are the size (SMB), value (HML), momentum (MOM), the [19]version of the original betting-against-beta factor (EWBAB), as well as their modified version (VWBAB), constructed using more standard procedures. VXO/VIX are the CBOE risk-neutral volatility indices. EMV is a text-based measure of uncertainty that tracks the equity market volatility, REG is a text-based measure of uncertainty that is constructed using news on aggregate regulation, FINREG is a text-based measure of uncertainty that is constructed using news on financial regulation and EPN is a text-based measure of uncertainty that is constructed using news on energy and environmental regulation.

## Results and discussions

My analysis can be motivated by taking the perspective of a mean-variance investor, which modifies its allocation in accordance with the attractiveness of the mean-variance trade-off.

**Fig 2:**
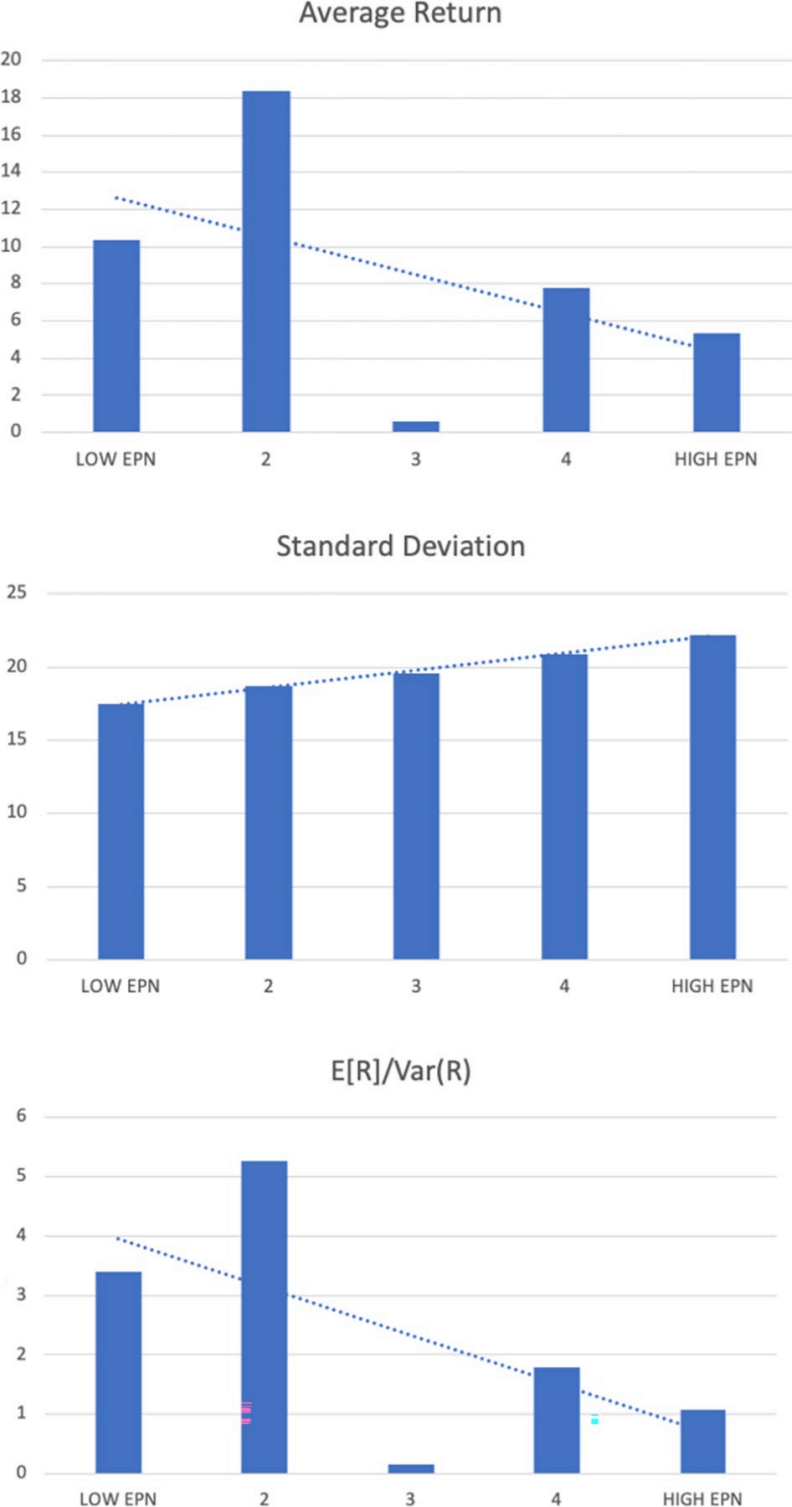
Sorts on the previous month’s policy-related uncertainty. In Fig 2, I use the monthly time series of the environmental-policy-news-related uncertainty indicator in the previous month to sort the following month’s returns into five buckets. E.g. the lowest, “LOW EPN”, looks at the properties of returns over the month following the lowest 20% of EPN-related uncertainty months. I show the average return over the following month, the standard deviation over the following month, and the average return divided by variance together with the corresponding trendline.

In Fig 2, I categorize months according to EPN-related uncertainty from the prior month and then compute the average returns, volatility, and mean-variance trade-off for the next month. Average return per unit of variance represents both "effective risk-aversion" from a general equilibrium perspective and the ideal level of risk exposure for a mean-variance investor in partial equilibrium. Average returns and lagged EPN uncertainty are strongly negatively correlated, although current volatilities and lagged EPN uncertainty are positively correlated. This indicates that when there is less EPN-related uncertainty, the mean-variance trade-off becomes stronger. This pattern suggests that a normal mean-variance investor should time EPN-related uncertainty by increasing risk when the mean-variance trade-off is favorable (low EPN-related uncertainty) and decreasing risk when the mean-variance trade-off is unfavorable (high EPN-related uncertainty).

Hence, assuming that environmental policy can be considered to be a noisy signal of future economic performance, I hypothesize that on average the relationship between EPN and expected returns is negative. The findings of [7] suggest that scaled portfolios produce positive alphas and increase Sharpe ratios. I make use of their methodology and use the EPN indicator in period t-1 to scale the monthly market factor returns in period t+1 in order to achieve a given target. One might argue that news-based measures rely on weekend news coverage not yet priced in the stock market. In line with [7], I tackle these concerns by skipping a month in the design of the trading rules. The scaled portfolio weight in the original market factor at time t+1 is given by $w_{t+1}=\frac{{EPN}_{target}}{{EPN}_{t-1}}$., In [21], among others, the authors argue that a target seems like a natural choice, as in the volatility-timing case, constant volatility is desirable to avoid the time-varying probability of having large losses in the portfolio. The choice of the target is arbitrary, but I always choose a target that produces scaled portfolio returns with the same ex-post downside risk compared to the (unscaled) market factor, where downside risk is defined as the 1-month, 5% Value-at-Risk using the historical simulation approach. In line with other studies, I find that my results are not sensitive to the choice of the target. Alternatively, I choose other VaR confidence levels or a target that produces scaled portfolio returns with the same ex-post volatility compared to the (unscaled) market factor. For example, in the latter case, the average weight $\frac{1}{n}\sum_{t=1}^{n}w_{t}$ over the sample period is 99.3%.

Given the low number of articles, there is a question of whether EPN really affects the equity return? One might argue that aggregate information related to various forms of regulation drives my results, and not necessarily specific news related to energy and environmental regulation. For example, the aggregate regulation or financial regulation category receive attention in around 25% or 15% of EMV articles, respectively. I replicate my analysis for both categories. Furthermore, asset pricing theory allowing for time-variation in risk premia assumes that options contain information that relate to fluctuations in expected equity market volatility and predicts that times of relatively high risks are followed by above average excess returns on the market portfolio (e.g. [12, 15]). Hence, I replicate my analysis using the VXO as a more traditional forward-looking risk measure and relying on a positive relationship between the VXO and expected return, assuming that option-implied volatility can be assumed to be a precise signal in the context of [7]. I use the older VXO as my option-implied risk measure for the early years and merge it with the VIX, because it grants me more data. However, the results don’t change if I only use the more recent VIX. In [3], the authors find that EMV moves with the VIX, hence I use the equivalent trading rule for the EMV. The proposed trading strategy makes use of the VIX (EMV) in period t-1 to scale the monthly market factor returns in period t+1 in order to achieve a given target in terms of forward looking volatility risk. The scaled portfolio weight in the original market factor of the VIX (EMV)-related trading strategy at time t+1 is given by $w_{t+1}=\frac{{VIX}_{t-1}}{{VIX}_{target}}(w_{t+1}=\frac{{EMV}_{t-1}}{{EMV}_{target}})$, respectively. Hence, I invest more (less) when past risks have been above (below) the target. Again, I choose targets that produce scaled portfolio returns with the same ex-post downside risk compared to the (unscaled) market factor, where downside risk is defined as the 1-month, 5% Value-at-Risk using the historical simulation approach.

**Fig 3:**
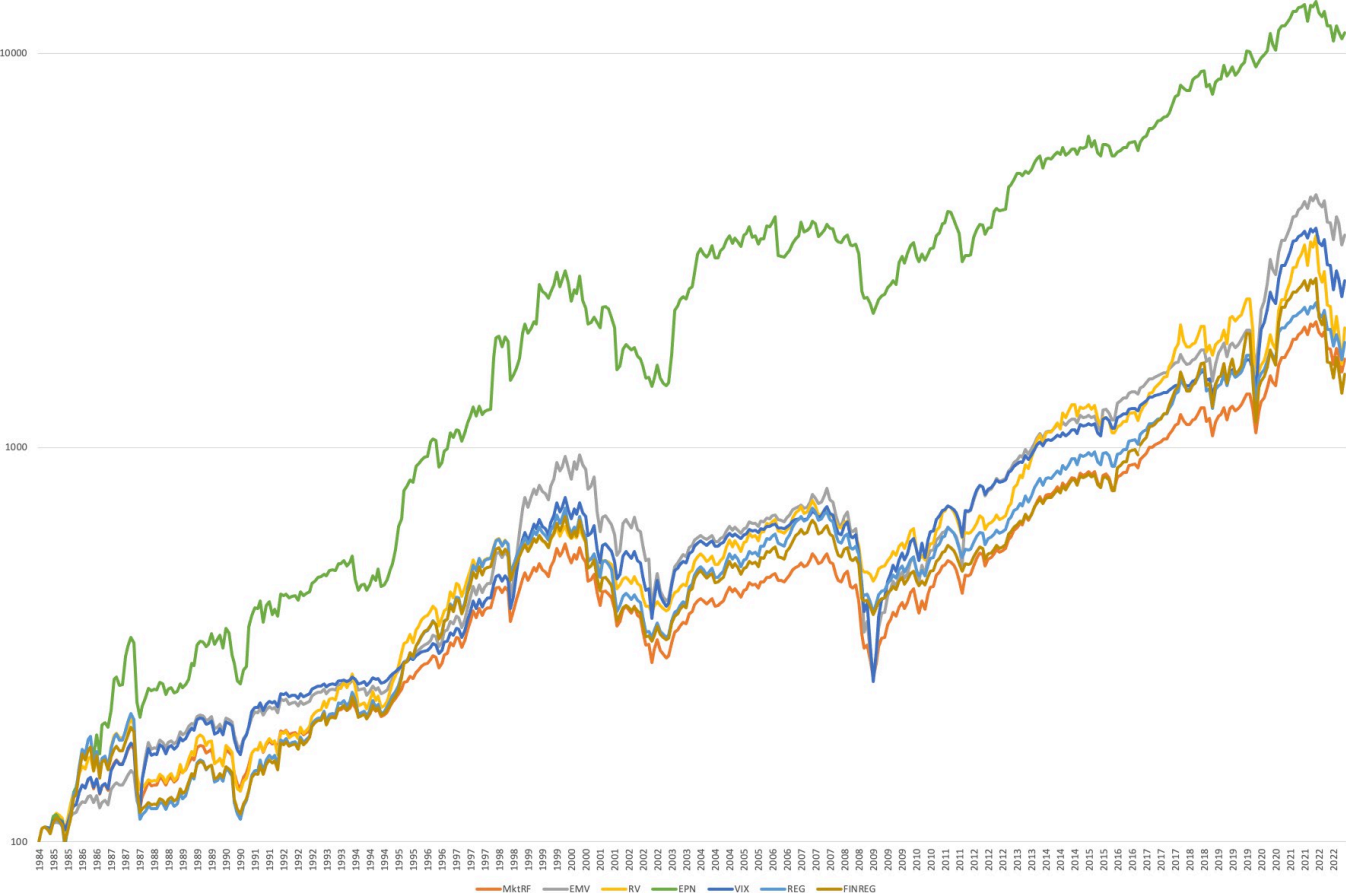
Performance of the EPN-managed trading strategy. The Fig presents the performance of the EPN-trading strategy vis à vis the other dynamic trading strategies based on $100 invested in January 1985 (logarithmic scale).

In Fig 3, I compare the EPN-managed market factor strategy with the original (unscaled) market factor returns and the alternative REG, FINREG, VIX and EMV-managed strategies, as well as the original Moreira and Muir-type [7] realized volatility-managed trading rule. I compute the realized equity market volatility at the end of each month using daily S&P500 index returns in the previous 20 days. Results suggest that the managed portfolios outperform the unscaled market factor portfolio. Interestingly, the EPN-managed portfolio outperforms the VIX (EMV)-managed market factor by 0.53% (0.56%) monthly, which is significant at the 1% level. Results also suggest that news related to aggregate regulation or financial regulation are not equally informative. As a result, my results are indeed driven by environmental policy news, hence, EPN dominates other forms of regulation.

While all strategies have the same ex-post downside risk, they might be differently exposed to other risk factors. In general, a time-series uncertainty- or volatility-based trading strategy might exploit an anomaly documented in the cross section. For example, research suggests that market volatility has strong predictive power for momentum returns. For example, in [22], the authors find that market volatility has significant power to forecast momentum profits, which is robust after controlling for market state and business cycle variables. Periods of low market volatility are followed by periods, where loser stocks significantly underperform winner stocks, resulting in high momentum profits. Furthermore, in [20], the authors show that a strategy that goes long low-beta stocks and shorts high- beta stocks (betting-against-beta or BAB) can earn large alphas relative to the CAPM and the Fama-French three-factor model that includes a momentum factor. Conceptually, my uncertainty—or volatility-based strategy are different, because the high risk-adjusted return of the BAB factor reflects the fact that differences in average returns are not explained by differences in CAPM betas in the cross-section, while the managed market strategies are based on the fact that across time periods, differences in average returns are not explained by differences in stock market variance. However, as with momentum, given its relation with market volatility, the BAB strategy might capture similar phenomena in the data. Hence, I also control for both BAB factors, the [19] version of the original BAB factor (EWBAB), as well as their modified version (VWBAB).

In Table 2, I present the equity risk-adjusted performance of the EPN-, the EMV- and the VIX- managed strategies. I report the CAPM-, the Fama-French three-factor- as well as the momentum and BAB alphas that are typically used in the literature. Results suggest that the volatility-managed strategies obtain insignificant alphas suggesting that they do not outperform the passive benchmark on a risk-adjusted basis. Indeed, the positive alpha for the volatility-managed market factor is driven by momentum. Interestingly, as hypothesized, I obtain a negative and statistically significant loading on momentum. Furthermore, I find that alphas are also impacted if I add the BAB factors as a control. Thus, the time-series volatility-managed portfolios are not completely distinct from the low-beta anomaly documented in the cross-section. In contrast, the EPN-managed strategy substantially outperforms the passive benchmark on a risk-adjusted basis. Hence, a managed equity portfolio that takes less (more) risk when EPN-related uncertainty is high (low) generates an annualized equity-risk-adjusted alpha of 5%. Interestingly, the R^2^ for the EPN-managed strategy is substantially lower compared to the volatility-managed counterparts further suggesting that traditional risk factors are not necessarily explaining its performance. The high betas of the strategies are because I am targeting ex-post downside risk. If the target is ex-post volatility, the CAPM beta for e.g. the EPN-managed strategy decreases to 0.80.

**Table 2:** Risk adjustment for MktRF-based strategies.

$X_{t}=\alpha +\beta_{1}{MktRF}_{t}+\beta_{2}{SMB}_{t}+\beta_{3}{HML}_{t}+\beta_{4}{MOM}_{t}+\beta_{5}EWBAB+\beta_{6}{VWBAB}_{t}+\epsilon_{t}$								
	a(%)	Mkt-Rf	SMB	HML	MOM	EWBAB	VWBAB	Adj. R^2^ (%)
*EPN-managed MktRf*	0.48***	1.12***						62.8
	(0.00)	(0.00)						
	0.47***	1.10***	0.03	-0.13**				63.1
	(0.00)	(0.00)	(0.59)	(0.03)				
	0.41**	1.12***	0.03	-0.10*	0.07			63.3
	(0.02)	(0.00)	(0.59)	(0.09)	(0.17)			
	0.40**	1.19***	0.05	-0.08	0.07	0.03		63.8
	(0.02)	(0.00)	(0.37)	(0.29)	(0.23)	(0.58)		
	0.40**	1.20***	0.07	-0.09	0.06		0.07	64.0
	(0.02)	(0.00)	(0.28)	(0.19)	(0.35)		(0.33)	
*EMV-managed MktRf*	0.12	1.18***						82.1
	(0.42)	(0.00)						
	0.15	1.18***	-0.01	-0.01				82.0
	(0.42)	(0.00)	(0.86)	(0.83)				
	0.20	1.15***	-0.01	-0.06	-0.12***			82.7
	(0.12)	(0.00)	(0.85)	(0.29)	(0.00)			
	0.19	1.13***	-0.00	0.11*	-0.07	-0.24***		83.6
	(0.16)	(0.00)	(0.93)	(0.09)	(0.13)	(0.00)		
	0.18	1.12***	-0.03	0.05	-0.08**		-0.15***	82.8
	(0.17)	(0.00)	(0.47)	(0.45)	(0.02)		(0.00)	
*VIX-managed MktRf*	0.06	1.12***						87.9
	(0.51)	(0.00)						
	0.05	1.12***	0.01	0.05				87.9
	(0.62)	(0.00)	(0.81)	(0.35)				
	0.13	1.09***	0.01	0.01	-0.11***			88.7
	(0.20)	(0.00)	(0.83)	(0.89)	(0.00)			
	0.15	1.07***	0.00	0.13*	-0.06	-0.16***		89.7
	(0.11)	(0.00)	(0.91)	(0.08)	(0.18)	(0.00)		
	0.11	1.06***	-0.01	0.08	-0.08**		-0.09***	89.1
	(0.28)	(0.00)	(0.67)	(0.25)	(0.03)		(0.00)	

The table presents the alphas and factor loadings from time-series regressions of the monthly returns of the dynamic trading strategies on equity risk factors. The full sample period ranges from January 1985 to October 2022, covering a total of 454 months. MktRF is the excess market return, the value-weighted returns of NYSE, Amex, and Nasdaq stocks from the Center for Research in Security Prices (CRSP) over 30-day T-bill return in %. Other factors are the size (SMB), value (HML), momentum (MOM) and the [19] version of the original betting-against-beta factor (EWBAB), as well as their modified version (VWBAB), constructed using more standard procedures. For each regression, the first row presents the coefficient estimates and the second row reports heteroskedasticity-adjusted p-values (in parentheses). ***, ** and * indicate statistical significance at the 1%, 5% and 10% level, respectively.

A plausible explanation for my results is the information precision hypothesis of [7]. In the context of a dynamic asset pricing model with a representative agent, the author shows that the relationship between expected returns and conditional volatility is ambiguous and depends on the precision of the information signal of future economic performance, hence, investors’ level of uncertainty. When investors’ uncertainty increases (the external signal is not precise), the conditional expected excess return may become negative. Hence, volatility driven by learning about structural parameters might be priced differently than volatility driven by standard forms of risk. Given that environmental policy can be considered an external imprecise signal, an increase in environmental policy news leads to an increase in investors’ uncertainty and, as a result, to negative expected returns. Hence, I hypothesize that, by taking less risk, the EPN-managed strategy outperforms a risk-based strategy (e.g. the EMV-managed strategy) when the signal appears in periods of high equity risk, e.g. high VIX and, presumably, high risk aversion. I run the following regression

$$r_{t+1}^{EPN}=\alpha_{0}+\alpha_{1}D_{t-1}+\beta_{0}r_{t+1}^{EMV}+\beta_{1}D_{t-1}r_{t+1}^{EMV}+\epsilon_{t+1},$$

which gives the relative beta of the EPN-managed market factor with respect to the EMV-managed market factor (EMV beta) conditional on past VIX being above a threshold (${VIX}_{t-1}>{VIX}_{high}$) compared to the unconditional estimate, where *D*_*t-1*_ is a high (top tercile) VIX dummy. I find that *β*_1_ is significantly smaller than 0, which suggests that the EPN-managed strategy takes less risk compared to the EMV-managed strategy during high VIX periods. The low VIX EMV beta of the EPN-managed market factor is 1.13 (t-stat 20.84) but the high VIX EMV beta coefficient is -0.73 (t-stat -10.17), making the EMV beta of the EPN-managed portfolio conditional on a high VIX period equal to 0.40. By taking less risk, the EPN-managed portfolio significantly outperforms (5% level) the EMV-managed market factor by 1.53% during high VIX periods, from 0.50% during low VIX periods. Hence, these findings suggest that the relationship between EMV or VIX and expected return is expected to be positive but observed to be negative due to the impact of environmental policy-related uncertainty.

Given the controversy around the contrasting approaches of Democrats and Republicans regarding energy and environmental regulation discussed in the introduction, I further test the differences of the EPN-managed portfolio conditional on one of the political parties having the majority in senate. Data is obtained from the History, Art & Archives website of the US House of Representatives. It provides information about the party divisions in both chambers of Congress and the party control of the White House. During my sample period, the party division in the senate was roughly 50:50. Overall, I find that the uncertainty around environmental policy is lower during periods when the Republicans have the majority. The average of the EPN measure in these periods is 26.3 compared to 30.8 during periods when the Democrats have the majority in senate, which is significantly different at the 1% level. In line with the previous analysis, given the lower uncertainty, I would expect the EPN-strategy to perform particularly well in periods when the Republicans have the majority in senate. In order to understand the magnitude of this effect, I run the following regression

$$r_{t+1}^{EPN}=\alpha_{0}+\alpha_{1}D_{Rep,t-1}+\beta_{0}{MktRF}_{t+1}+\beta_{1}D_{Rep,t-1}{MktRF}_{t+1}+\epsilon_{t+1},$$

which gives the relative beta of the scaled factor conditional on the Republicans having the majority in senate (*D*_*Rep*,*t*−1_ = 1) compared to the unconditional estimate. I find that the “Republicans market beta” is significantly higher compare to the “Democrats market beta”, which suggests that the EPN-managed strategy takes more risk during periods when the Republicans have the majority in senate. The Democrats market beta of the EPN-managed market factor is 1.04 (t-stat 18.09) but the Republicans market beta coefficient is 0.15 (t-stat 2.51), making the beta of the EPN-managed portfolio conditional on a Republicans period equal to 1.19. By taking more risk in a low EPN market, the monthly CAPM alpha increases to 0.66% during Republicans periods, from 0.15% during Democrats periods. In line with previous evidence, I further analyze two interesting sub-periods, which represent important episodes regarding expected shifts in US environmental policy: periods when President Clinton and President Obama took office after the 1992 and 2008 US presidential election, respectively. I focus on their first two years in office, when the democrats still had the majority in senate. Results suggest that the average of the EPN measure in these periods is significantly higher, 33.2 compared to 26.3 otherwise, and the market beta of the EPN-managed market factor is significantly lower in these periods. By taking less risk in a high EPN market, the monthly CAPM alpha decreases to 0.06% during these periods, from 0.46% otherwise. Apparently, in contrast to the good intentions, the environmental policy efforts of the Democrats create uncertainty among financial market participants.

## Conclusions

The information quality hypothesis of [7] suggests that the relationship between expected returns and conditional volatility is ambiguous and depends on the precision of the information signal of future economic performance, hence, it is affected by investors’ level of uncertainty. When investors’ uncertainty increases, the relationship may become negative. Using environmental policy as an imprecise signal, I find that a newspaper-based environmental policy-related uncertainty indicator has a low correlation with equity market volatility, but has a significant negative impact on expected returns. A managed equity market portfolio that takes less (more) risk when the past EPN-related uncertainty is high (low) produces significant equity-risk-adjusted alphas. In particular, I show that EPN-timing is profitable, because it foresees the attractiveness of the mean-variance trade-off. In other words, after periods of low EPN-related uncertainty, a mean-variance investor can achieve a large return by taking more risk. Overall, a managed equity portfolio that takes less (more) risk when EPN-related uncertainty is high (low) generates an annualized equity-risk-adjusted alpha of 5–6%. Interestingly, I find that the uncertainty around environmental policy is lower and, therefore, the strategy performs better during periods when the Republicans control the senate.
